# Differential Relationships between Locus Coeruleus Integrity and Regional Neural Activity between Young and Older adults

**DOI:** 10.1002/alz70861_108846

**Published:** 2025-12-23

**Authors:** Ji‐Hyun Kim, Xiaoyang Hu, Jasmine Ranieri, Hwamee Oh

**Affiliations:** ^1^ Brown University, Providence, RI USA

## Abstract

**Background:**

The locus coeruleus (LC), a major norepinephrine‐producing region, plays a central role in arousal and cognition functions. Understanding how the LC exerts its impact on cognition is of paramount importance especially given the region has been known to accumulate tau pathology even in young adulthood, preceding broad tau and amyloid deposition in the brain. Here, we examined the relationships between LC integrity and spontaneous regional brain activity across the adult lifespan to test the hypothesis of the role of LC‐related regional brain activity in cognition.

**Method:**

Forty‐nine cognitively normal adults (22 older (mean age: 70.7.44; 11 females) and 27 young (mean age: 25.5.81; 21 females)) from Brown Multimodal Imaging of Cognition, Aging, and Alzheimer’s Disease (MICAAD) study completed extensive neuropsychological tests and resting‐state T2* blood‐oxygenation‐level‐dependent (BOLD) MRI (rsfMRI) and LC MT‐MRI T1 imaging. LC intensity was calculated by normalizing the LC signal relative to the pontine tegmentum and five highest intensity voxels from each hemisphere were averaged to mean LC intensity values. Resting‐state fMRI data were preprocessed and denoised using nuisance variables, including the Friston 24 parameters as well as white matter and cerebrospinal fluid signals, to compute regional brain activity level implemented in the RESTplus v1.31 toolbox. Whole‐brain voxel‐wise analyses were conducted to examine LC intensity‐ and age‐related differences in regional brain activity across age groups, controlling for sex.

**Result:**

Across all participants, higher LC intensity was related to lower regional brain activity in widespread brain regions including the dorsomedial prefrontal cortex and the cerebellum. Age was mostly associated with lower regional brain activity, while activity in the left Insular cortex and right amygdala increased with advanced age. Within each age group, higher LC intensity was related to lower regional activity in the putamen and cerebellum among young, but not older adults, while older age was associated with greater dorsolateral PFC activity among older, but not young adults.

**Conclusion:**

LC intensity relates to regional brain activity differently across age groups. This differential relationships between LC intensity and regional brain activity by age groups may underlie a potentially different role of LC intensity in cognition across the lifespan.

